# The diagnostic performance comparison between T2 mapping and Dixon against the activity of thyroid-associated ophthalmopathy: a systematic review and meta-analysis

**DOI:** 10.3389/fendo.2024.1502296

**Published:** 2024-12-12

**Authors:** Fuyi Zhang, Pengcheng Wang, Chun Cao, Xinyu Pan, Tao Zhang, Meng Fan, Yu Guan

**Affiliations:** ^1^ Department of Ophthalmology, The Second Affiliated Hospital of Chengdu Medical College, Nuclear Industry 416 Hospital, Chengdu, Sichuan, China; ^2^ Department of Clinic Medicine, School Of Clinic Medicine, Chengdu Medical College, Chengdu, Sichuan, China; ^3^ Department of Biochemistry and Molecular Biology, School of Biological Sciences and Technology, Chengdu Medical College, Chengdu, Sichuan, China; ^4^ Department of Pathology and Pathophysiology, School of Basic Medical Science, Chengdu Medical College, Chengdu, Sichuan, China

**Keywords:** thyroid-associated ophthalmopathy, Dixon, T2 mapping, diagnosis, meta-analysis

## Abstract

**Objective:**

The aim of this study was to compare the diagnostic performance of T2 mapping and Dixon in thyroid-associated ophthalmopathy’s disease activity.

**Methods:**

Published studies were collected by systematically searching the databases PubMed, Embase, Cochrane Library, Google Scholar, Medline, Web of Science, CNKI, VIP, and WANFANG. The sensitivities, specificities, likelihood ratios, and diagnostic odds ratio (DOR) were confirmed. The symmetric receiver operator characteristic curve (SROC) was used to assess the threshold of T2 mapping and Dixon. Fagan’s nomogram was drawn. Meta-regression and subgroup analyses were applied to distinguish the sources of heterogeneity among the included studies. The review was conducted in accordance with the Preferred Reporting Items for Systematic Reviews and Meta-Analyses (PRISMA) 2020 statement.

**Results:**

A total of 17 studies were included, comprising 1,455 participants. The combined sensitivity of T2 mapping was 0.70 [95% CI (0.65–0.75)], specificity was 0.84 [95% CI (0.75–0.90)], area under the SROC curve (AUC) was 0.78 [95% CI (0.75–0.82)], and DOR was 12. The combined sensitivity of Dixon was 0.74 [95% CI (0.58–0.85)], specificity was 0.80 [95% CI (0.58–0.93)], AUC was 0.83 [95% CI (0.80–0.86)], and DOR was 11.66. The Deeks’ funnel plot showed no existing publication bias. The prospective design, partial verification bias, and blinding contributed to the heterogeneity in specificity and sensitivity. The post-test probability of T2 mapping in TAO patients’ disease activity was 75%, and the post-test probability of Dixon in TAO was 87%.

**Conclusion:**

Compared with T2 mapping, Dixon presented a significantly higher sensitivity and AUC for detecting TAO disease activity. Dixon is expected to further improve the accuracy of diagnosis of TAO’s disease activity.

## Introduction

Graves’ ophthalmopathy (GO), also called thyroid-associated ophthalmopathy (TAO), is mainly characterized by proptosis, upper eyelid retraction, edema, and diplopia and is described as an ocular autoimmune disorder with complicated pathogenesis (1, 2). TAO is the most common orbital disease, and women had a higher incidence (8.9 cases/100,000 person-years) than men (1 case/100,000 person-years) (3, 4). It can lead to significant ocular symptoms, facial disfigurement, vision loss, and decreased quality of life (5). The assessment of TAO activity and symptom severity is the basis for formulating treatment plans, and patients in the active phase require early anti-inflammatory treatment. Therefore, timely and accurate staging is crucial in clinical practice.

In 1989, Mouritis et al. (6) proposed the use of the clinical activity score (CAS) as a common clinical method for assessing the activity of TAO, with the drawback of being overly subjective and limited (7). Tachibana Seigo’s study indicated that orbital magnetic resonance imaging (MRI) combined with CAS can improve the sensitivity of detection of disease activity and prediction of response to immunosuppressive therapy for GO (8). As an effective method, MRI can provide a variety of structural and pathological information, offering objective imaging indicators for accurate evaluation. This method can effectively prevent various injuries caused by ionizing radiation and enhance the soft tissue resolution. Unfortunately, studies that compare the ability of various MRI techniques to assess the activity stages of TAO, as well as studies focusing on combined assessments, were limited. A precise and comprehensive unified quantitative evaluation standard has not yet been established.

T2 mapping used the multi-echo spin-echo pulse sequences to obtain a complete T2 decay curve composed of different time points along with multiple echoes (9). The T2 relaxation time (T2RT) derived from T2 mapping represents the decay rate of the magnetic resonance signal, which is a physical property of a tissue. Obviously, it is widely recognized as the objective value (10). This method reflects the water content in tissues. It is simple, objective, and accurate, and thus has gradually been applied to various diseases (11–15). Several studies have validated the great potential of T2 mapping technology in predicting active TAO patients (16–18).

Furthermore, Dixon is a fat-suppression technique based on chemical shift analysis, allowing the effective separation of water and fat (19, 20). The main feature of TAO is inflammatory infiltration and the remodeling of retrobulbar tissue. The Dixon sequence performs well for quantitative measurements of the orbital fat content and the edema degree of the extraocular muscles (EOM). The superiority of the Dixon technique to conventional inversion recovery or spectral presaturation in terms of overall image quality and FS uniformity has been fully reported (21–23).

However, few studies were performed to compare the diagnosis performance between T2 mapping and Dixon against the TAO disease activity. Consequently, this study mainly evaluates and compares the diagnostic value between nuclear magnetic resonance quantitative technology T2 mapping and Dixon for the activity of TAO.

## Methods

### Search strategy

Two reviewers (ZFY and WPC) searched PubMed, Embase, Cochrane Library, Google Scholar, Medline, Web of Science, CNKI, VIP, and WANFANG databases up to August 2024 independently. The T2 mapping search terms were as follows: [(T2 mapping) OR (T2RT) OR (T2 value) OR (MRI T2) OR (T2 relaxation time)] AND [(Graves disease) OR (TAO) OR (TED)]. The Dixon search terms were as follows: [(Graves disease) OR (Graves Orbitopathy) OR (TAO) OR (TED)] AND [(DIXON) OR (fat-suppression) OR (fat suppression)]. TAO, thyroid-associated ophthalmopathy; TED, thyroid eye disease.

### Inclusion and exclusion criteria

The inclusion criteria included the following items (1): clinical diagnosis TAO patients included as study subjects; (2) randomized controlled trials were divided into two groups: the experimental group with active TAO patients and the control group using patients with inactive TAO patients; (3) clinical trials involving T2 mapping or/and Dixon for TAO detection; (4) data of true-positive (TP) cases, false-negative (FN) cases, false-positive (FP) cases, and true-negative (TN) cases and indicators of sensitivity (Se) and specificity (Sp) shown or figured out according to the literature; and (5) CAS grade was applied as the gold standard method of diagnosis. The exclusion criteria included the following items: (1) animal studies; (2) non-case–control trials; (3) studies without sufficient or experimental data; (4) letters, case reports, guidelines, reviews, and conference abstracts; (5) published literature repeatedly; and (6) unrelated studies to diagnostic means in TAO patients.

### Data extraction

Two researchers (ZFY and WPC) independently conducted data extraction from the studies and all disagreements were resolved by consensus with all investigators. The following data were extracted: study characteristics (region, year of publication, type of study, and sample size), patient characteristics (age, sex, presence of metabolic syndrome, and laboratory parameters), the gold standard (CAS) used and the outcome indicators of Dixon and T2 mapping, which included TP, FP, FN, TN, Sp, and Se.

### Quality assessment

The diagnostic experimental Quality Assessment of Diagnostic Accuracy Studies-2 (QUADAS-2) tool of the RevMan5.3 software was used to evaluate the quality of the included literature and assess the risk of bias and applicability of each included literature (24). Each study was evaluated for risk of bias and applicability following four key domains: patient selection, index test, reference standard, and flow and timing. There were two high risk and six unclear risk in patient selection, five high risk and two unclear risk in index test, and three high risk and five unclear risk in flow and timing. Disagreements were resolved by consensus.

### Statistics

The diagnostic modalities of studies were analyzed by Stata software (version 15.0). The bivariate model was used to calculate combined sensitivity, specificity, the positive/negative likelihood ratio (PLR/NLR), and diagnostic odds ratio (DOR). The area under the receiver operator characteristic (ROC) curve estimated the total diagnostic efficacy of Dixon or T2 mapping in Tao patients’ disease activity. The pre-test probability was assessed from conventional data, trial data, or clinical decisions. Post-test probability could determine whether diagnostic probability increased or reduced compared to pre-test probability. The statistical heterogeneity based on the included studies was evaluated using the *I* ^2^ statistics and *Q* test. Values of *I* ^2^ < 50% and *p* > 0.1 indicated what could be regarded as inhomogeneity; thus, a random-effects model was applied for further analysis. Otherwise, a fixed-effect model should be performed. A *p*-value <0.05 indicated a significant difference.

## Results

### Flowchart and study quality

**Figure 1:**
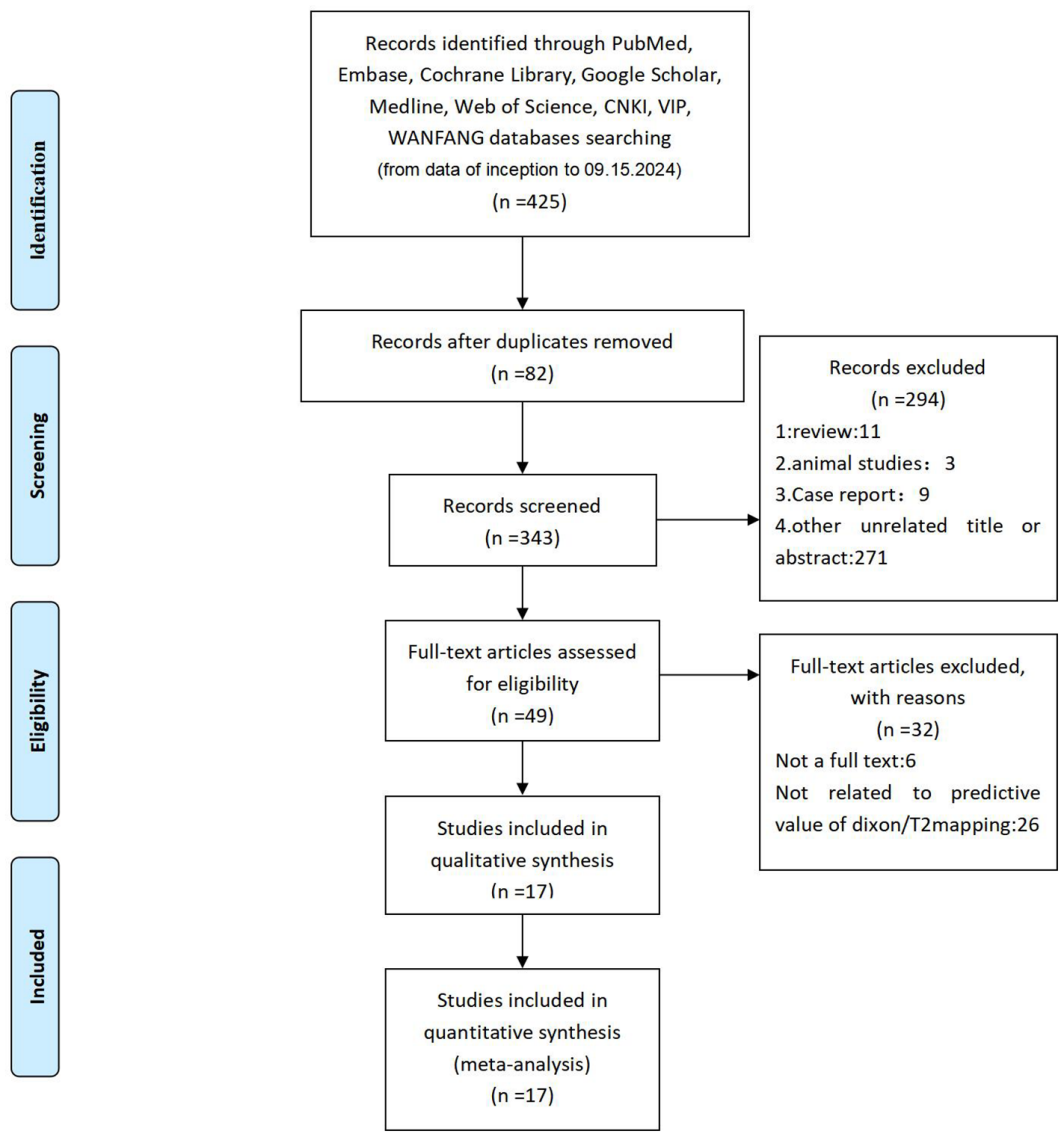
Literature screening process of the meta-analysis.

A total of 425 studies (including documents, reviews, animal experiments, case reports, and repeated studies) were retrieved from each database. After utilizing Endnotes software and manually removing 82 articles based on duplicate titles and abstracts, 343 relevant studies were included. Among these studies, 23 were excluded for being reviews, meta-analyses, or case reports, while 271 studies did not have related titles and abstracts. The full text of the remaining 49 studies was selected, and 32 studies were removed after reading the full text due to incomplete data; for example, the information on Sp, Se, or AUC was missing. The remaining 17 studies were extracted from the corresponding data according to the data extraction requirements. A total of 11 studies used T2 mapping, and 6 used Dixon. We followed the Preferred Reporting Items for Systematic Reviews and Meta-Analyses (PRISMA) (25), and the literature screening process is shown in **Figure 1**. The basic characteristics of each study are plotted in **Table 1**.

**Table 1:** Basic characteristics of enrolled studies.

Reference	Year	Region	Sample size	Scale used to assess dysfunction	Diagnostic value		
					Sensitivity (%)	Specificity (%)	AUC
Lu Wang (26)	2024	China	49	T2 mapping	93.8	80	0.925
Qing Zhang (27)	2020	China	92	T2 mapping	72.9	70	0.821
Qing Zhang (27)	2020	China	92	T2 mapping	80	65.4	0.907
Defu Li (28)	2021	China	42	T2 mapping	63.5	90.09	0.8
Hong Jiang (29)	2018	China	74	T2 mapping	48.15	97.65	0.75
Zhangfang Li (30)	2023	China	235	T2 mapping	75	93.8	_
Jingyi Cheng (31)	2023	China	68	T2 mapping	70.7	69.3	0.745
WEN CHEN (32)	2019	China	36	T2 mapping	79.4	93.3	0.863
WEN CHEN (33)	2020	China	32	T2 mapping	73.3	80	0.868
Luyan Su (34)	2022	China	98	T2 mapping	92.2	58.3	0.747
Libin Yang (35)	2024	China	56	T2 mapping	86	88	0.882
Defu Li (36)	2024	China	44	T2 mapping	63.5	90.09	0.92
Alexis Ollitrault (37)	2020	France	206	Dixon	100	71	_
Xiong-Ying Pu (38)	2024	China	200	Dixon	84.02	66.89	0.82
Kai Huang (39)	2023	China	70	Dixon	65.9	94.5	0.865
Xiaoting Feng (40)	2020	China	66	Dixon	56.1	80.68	0.696
Lu Chen (23)	2020	China	37	Dixon	75	85.3	0.86
Gang Liu (41)	2019	China	50	Dixon	74.14	66.67	0.703

### T2 mapping against the TAO

**Figure 2:**
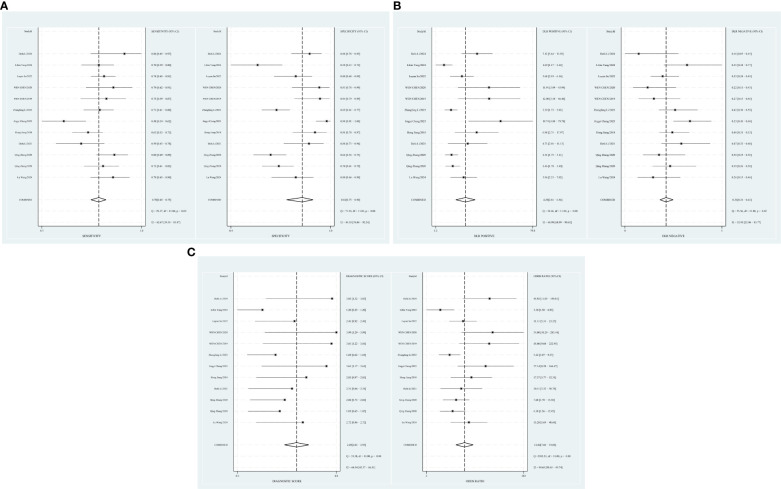
**(A)** Forest plot of sensitivity and specificity of T2 mapping in the diagnosis of TAO. **(B)** Forest plot of diagnosis likelihood ratio (DLR) of T2mapping in the diagnosis of TAO. **(C)** Forest plot of the diagnostic odds ratio (DOR) of T2mapping in the diagnosis of TAO.

The combined sensitivity of T2 mapping against the TAO was 0.70 [95% CI (0.65–0.75)], specificity was 0.84 [95% CI (0.75–0.90)], PLR was 4.3 [95% CI (2.8–6.5)], NLR was 0.36 [95% CI (0.31–0.41)], and DOR was 12, indicating that T2 mapping had a moderate value in the screening of TAO. The random-effects model was applied because the heterogeneity was greater than 50%. For more details, please see **Figures 2A–C**.

### Publication bias and heterogeneity

The Deeks’ funnel plots were used to assess potential publication bias in detecting TAO with T2 mapping. As shown in **Supplementary Figure 1**, publication bias existed, with a *p*-value of 0.01. The bivariate boxplot showed that two studies were out of the circles, indicating heterogeneity between included studies, as shown in **Supplementary Figure 2**.

### Threshold effect

**Figure 3:**
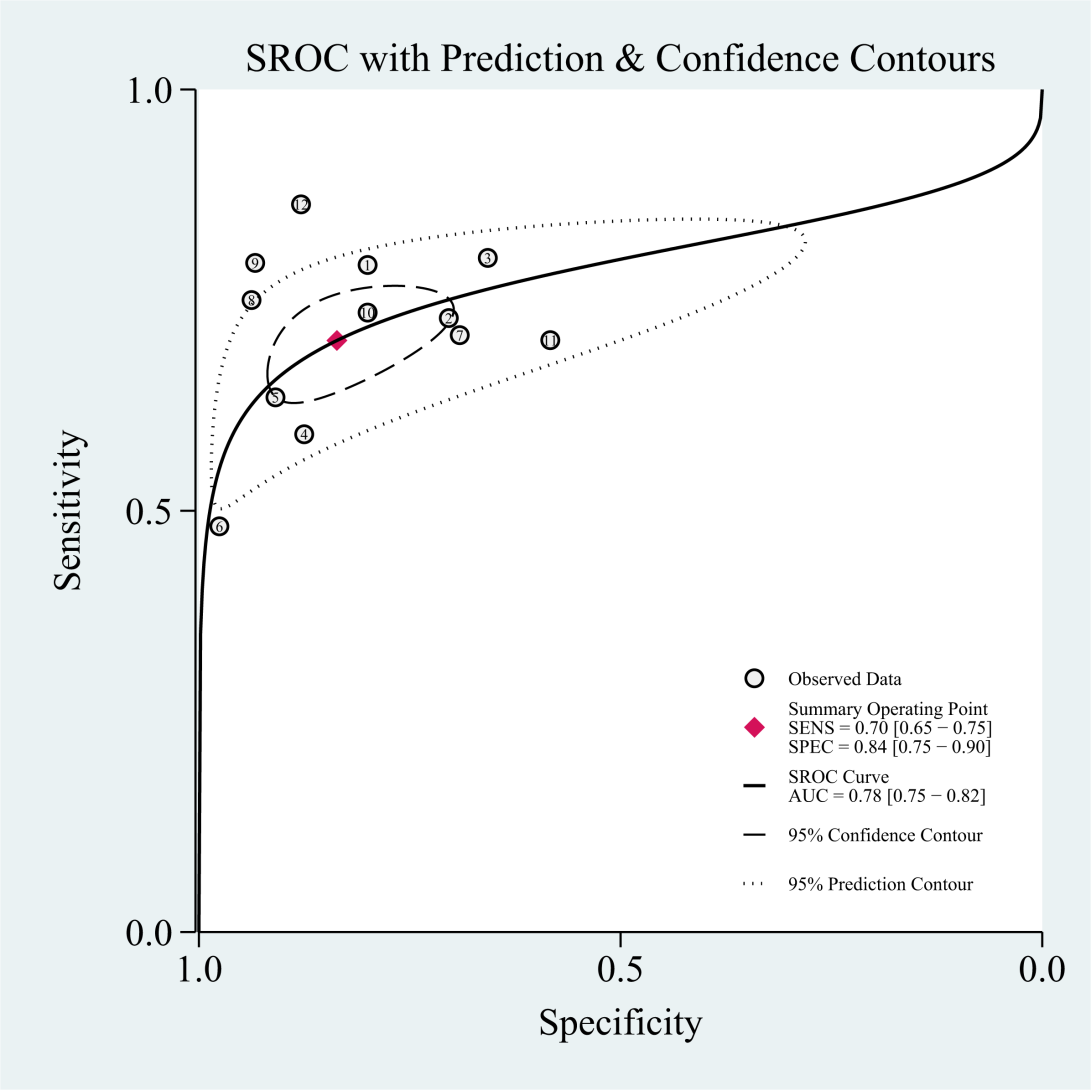
Summary of receiver operating characteristics of T2 mapping.

The threshold effect was assessed by the SROC curve plane test. **Figure 3** shows the absence of the typical “shoulder arm”, representing the inexistence of the threshold effect. This implies that there is no apparent trend of sensitivity that first increases and then decreases with specificity across different thresholds. Then, the differences in sensitivity and specificity among different studies can be attributed primarily to factors such as study design, sample size, and detection methods, rather than being caused by variations in the threshold. The area under the SROC curve (AUC) was 0.78 [95% CI (0.75–0.82)], indicating a moderate diagnostic value of T2 mapping.

### Pre-test probability, LR, and post-test probability

**Figure 4:**
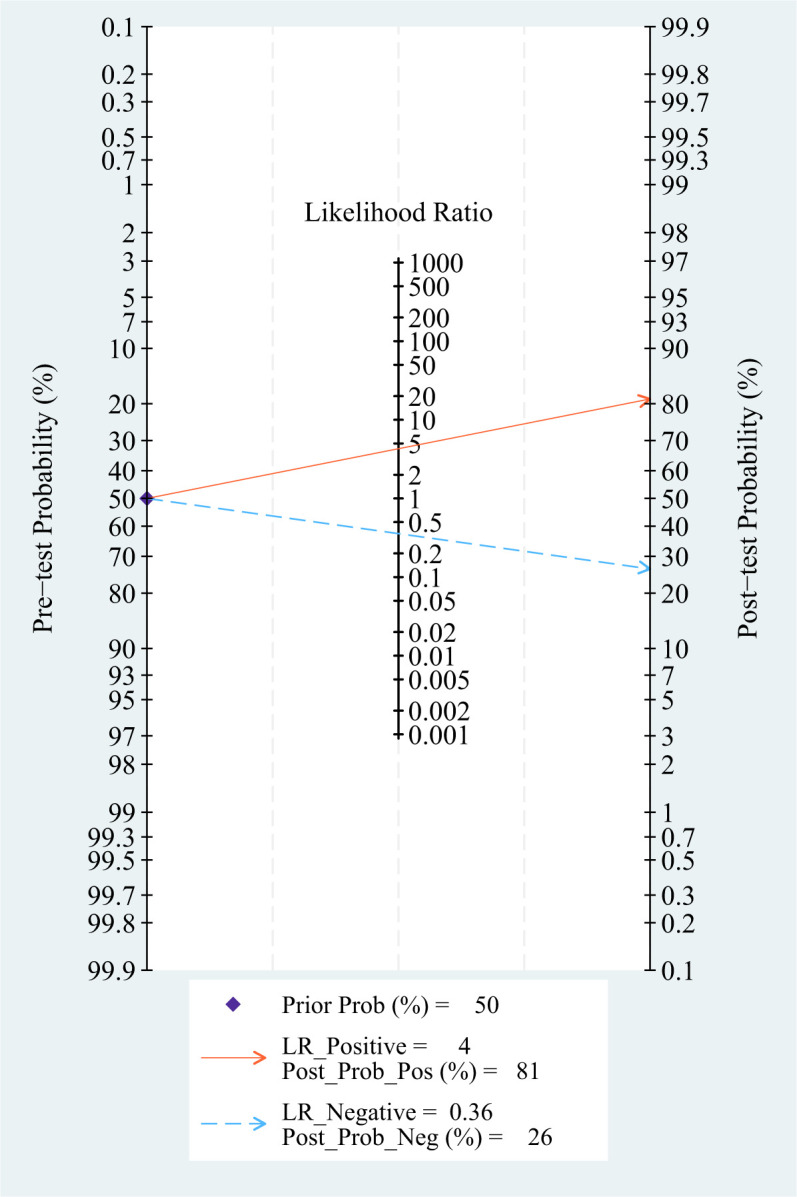
Fagan diagram of T2 mapping in the diagnosis of TAO.

The relationship among the prior probability, the PLR, the NLR, and the posterior probability were performed in the Fagan graph. The post-test probabilities were calculated using the Stata software. Setting the pre-test probability as 50% previously, the post-test probability of TAO was 75%. Moreover, the positive likelihood ratio (PLR) was less than 10 (PLR = 4), and the negative likelihood ratio (NLR) was >0.1 (NLR = 0.36), indicating that the diagnosis could neither be confirmed nor excluded (see **Figure 4**).

### Meta-regression and subgroup analysis

Some factors, including prospective design (prodesign), partial verification bias (fulverif), and adequate description of study participants (subjdescr), are reported, and whether the test results were assessed by a blind method might be relevant to heterogeneity among these T2 mapping studies. The meta-regression analysis of the above-mentioned factors indicated that prodesign, fulverif, and blind could affect the heterogeneity of sensitivity, but less affect the heterogeneity of specificity, as plotted in **Supplementary Figure 3**.

### Dixon against TAO

**Figure 5:**
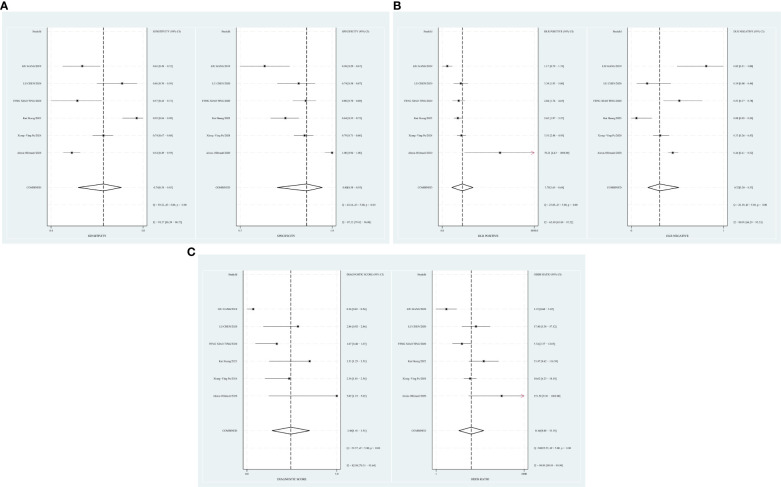
**(A)** Forest plot of sensitivity and specificity of Dixon in the diagnosis of TAO. **(B)** Forest plot of diagnosis likelihood ratio (DLR) of Dixon in the diagnosis of TAO. **(C)** Forest plot of the diagnostic odds ratio (DOR) of Dixon in the diagnosis of TAO.

A random-effects model was applied when the heterogeneity was greater than 50%. The combined sensitivity of Dixon assessing the disease activity of the TAO patients was 0.74 [95% CI (0.58–0.85)], specificity was 0.80 [95% CI (0.58–0.93)], PLR was 3.78 [95% CI (1.65–8.68)], NLR was 0.32 [95% CI (0.20–0.53)], and DOR was 11.66, indicating that Dixon had a moderate value in the assessment of the TAO patients’ disease activity (**Figures 5A–C**).

### Publication bias and heterogeneity

A *p*-value of 0.20 (*p* > 0.05) (**Supplementary Figure 4**) indicated the absence of publication bias. There was one study outside of the border, representing heterogeneity among the included studies (see **Supplementary Figure 5**).

### Threshold effect

**Figure 6:**
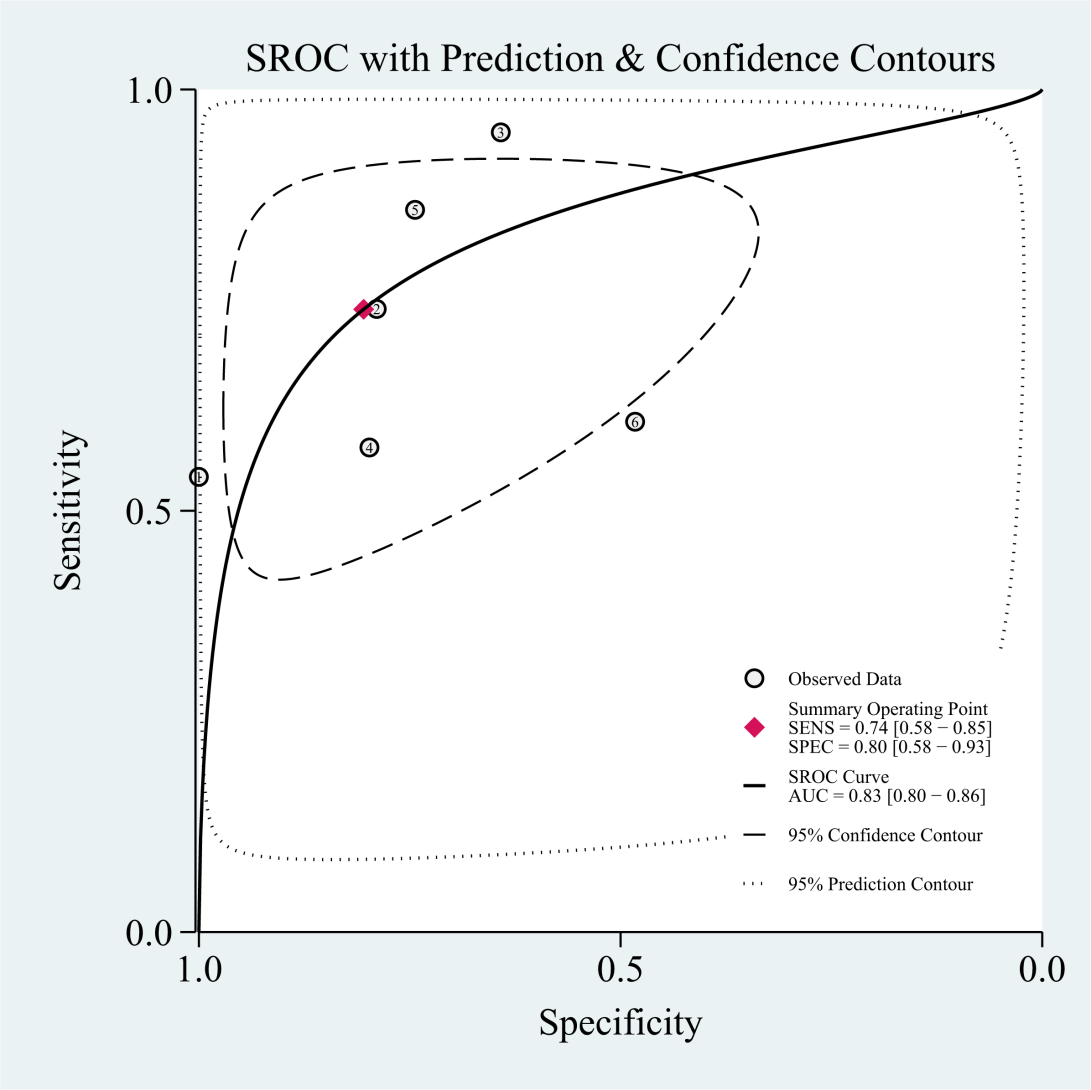
Summary of receiver operating characteristics of Dixon.

The threshold effect was assessed by the SROC curve plane test. The typical “shoulder arm” was not revealed in **Figure 6**, representing the inexistence of a threshold effect. The AUC was 0.83 [95% CI (0.80–0.86)], indicating a moderate diagnostic value of Dixon.

### Pre-test probability, LR, and post-test probability

**Figure 7:**
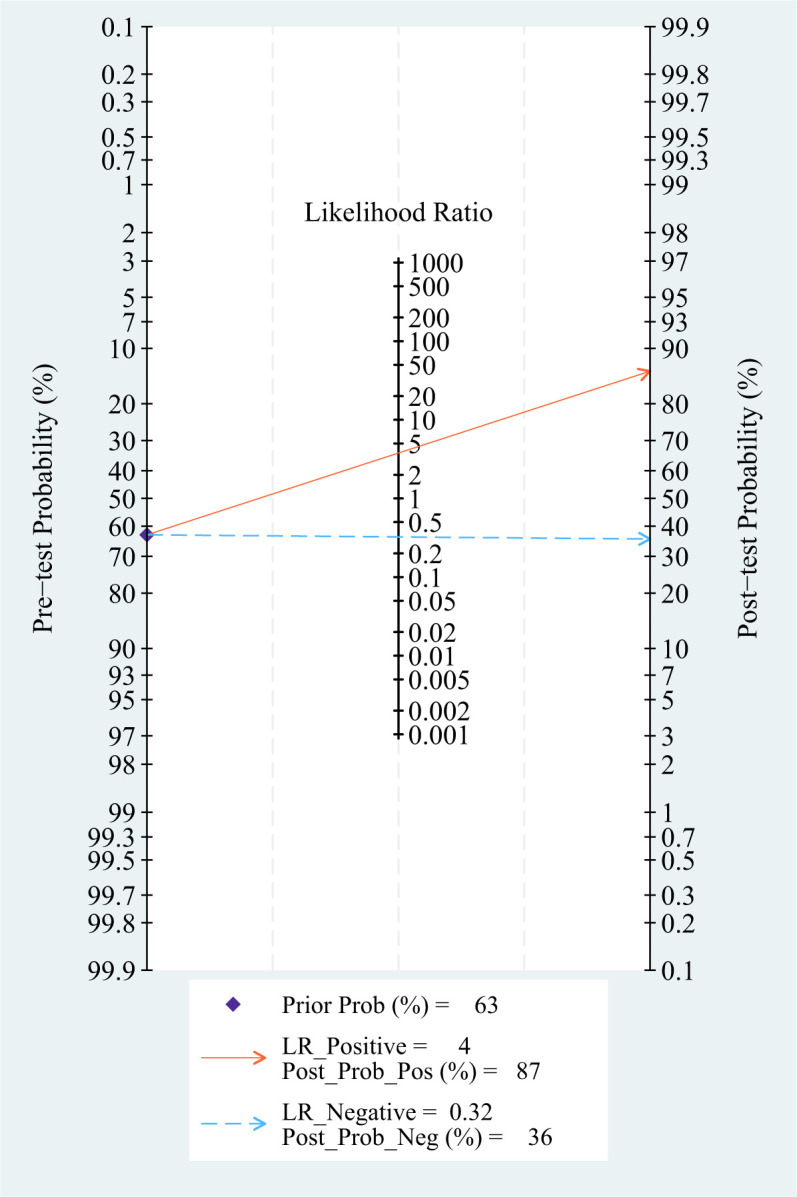
Fagan diagram of Dixon in the diagnosis of TAO.

Setting the pre-test probability as 63% in advance, the post-test probability of TAO patients was 87%. The post-test probabilities were also calculated. Moreover, the PLR was less than 10 (PLR = 3.8), and the NLR was greater than 0.1 (NLR = 0.32). The value of diagnosis and excluded of Dixon against TAO disease were both limited (see **Figure 7**).

### Meta-regression and subgroup analysis

The meta-regression analysis indicated that the factors including fulverif are blinded and did not affect the heterogeneity of sensitivity and specificity (see **Supplementary Figure 6**).

### Comparison of T2 mapping and Dixon

The comparison between T2 mapping and Dixon was demonstrated by ROC, sensitivity, and specificity analyses. Between T2 mapping and Dixon, the latter presented a better diagnostic value among AUC, sensitivity, and specificity (see **Table 2** for details).

**Table 2:** Diagnostic performance of T2 mapping and Dixon.

Method	AUC	Sensitivity	Specificity	Prior *p*	PLR	NLR
T2 mapping	0.78	0.7	0.84	0.50	4.3	0.36
Dixon	0.83	0.74	0.80	0.63	3.78	0.32

## Discussion

Currently, there is still a lack of accurate diagnostic methods for TAO activity. Early diagnosis can significantly improve treatment. Therefore, the diagnosis of TAO activity is of great clinical significance. This systematic review and meta-analysis assessed the diagnostic efficiency of T2 mapping and Dixon in TAO. In brief, 17 studies were included, involving 1,455 samples. Two diagnostic methods have moderate value for DOR as an active diagnosis for evaluation. Meanwhile, Dixon has a higher sensitivity and higher ROC than T2 mapping. Studies have found that many patients with a CAS of 1 or 2 show a significant response to immunosuppressive therapy, and they suggest that the CAS cutoff of 3 points as stated by the European panel may not be appropriate for Asian populations (8). Thus, finding a valuable index to distinguish the stage of TAO patients in Asian populations remains a great challenge.

Dixon can quantitatively measure the water–fat content of tissues. It presents the advantages of short scanning time and good fat suppression. CAS is often used to evaluate the activity of TAO. However, CAS has strong subjectivity, low sensitivity, and low specificity. For example, Son’s study showed that all but one water map equals the fat suppression sequence. Dixon-T2WI can also generate fat maps, allowing quantitative analysis of fat content, and with higher signal values in the edematous fraction, Dixon-T2WI was shown to improve the sensitivity and specificity of the diagnosis (42). Regarding Dixon, six studies that exhibited heterogeneity in diagnosing the activity of TAO patients due to their different choice of effect measures were included. Kai Huang (39) and Lu Chen (23) utilized the signal intensity ratio of extraocular muscles (SIR-EOM). Liu Gang (41) selected the fat fraction (FF) as his parameter. Feng Xiaoting (40) incorporated both SIR-EOM and FF in her analysis. Alexis Ollitrault (37) chose EOM inflammation as his primary parameter. On the other hand, Xiong-Ying Pu (38) combined the EOM-SIR, the Lacrimal Glands-SIR (LG-SIR), and the LG-FF. Among these, the study by Alexis Ollitraul (37) and his team exhibited the highest sensitivity of 100%, which may be attributed to its prospective nature, the inclusion of 206 patients, and the implementation of a second reading session for imaging analysis 8 weeks later to assess intra-observer agreement. Conversely, Feng Xiaoting’s study (40) had the lowest sensitivity of 56.1%, which may be related to the lack of blinding and consistent validation methods. In Liu Gang’s study (41), the small sample size may have inevitably led to bias in data collection.

T2 mapping is a quantitative MRI technique. T2RT is tissue-specific, which can reflect the subtle changes of disease evolution and treatment, and achieve non-invasive quantification of histopathological changes. For example, Luo’s study disclosed that by using the T2RT, orbital MRI not only detects the presence or absence of swollen tissue, but also objectively and quantitatively evaluates the inflammatory activity of the orbital tissue in TAO patients (43). In T2 mapping, we included 11 studies, namely, 5 retrospective studies and 6 prospective studies. Each study included a large number of subjects and had a satisfactory description of the indicators, a statistical description of the trial, an adequate description of the study subjects, satisfactory reporting of the results, and strict design and execution criteria. However, only five articles mentioned the use of blinding, and three articles did not use the same method of verification.

Both T2 mapping and Dixon had a similar diagnostic performance for TAO, with a DOR of 12 and 11.66, respectively. Dixon was slightly superior to T2 mapping in the diagnosis of active TAO (0.74 vs. 0.70). The diagnostic efficacy of T2 mapping in the diagnosis of inactive patients was slightly higher than that of Dixon (0.84 vs. 0.80), but the AUC under the ROC curve of Dixon was slightly higher than that of T2. In clinical practice, Dixon is mainly used for the evaluation of liver fat deposition and breast MRI to eliminate the interference of high fat signal, inflammation, and edema. The basic pathological features of TAO include the infiltration of immune cells in the orbit, the deposition of hydrophilic substances, and the enlargement of EOM and orbital adipose tissue, and the main pathological changes in the active stage are the infiltration of inflammatory cells and inflammatory edema in the orbital tissue. Therefore, Dixon presented much better diagnostic efficiency than T2 mapping.

Dixon and T2 mapping also have their own limitations in the terms of diagnostic value. At present, T2 mapping technology is mainly used to judge the activity of EOM. There are few studies on the judgment of lacrimal gland and orbital fat that need to be further validated. The Dixon technique is mainly based on long echo sequences, and it is sensitive to motion. It has certain limitations in TAO patients, whose eyeballs could not remain still during the examination.

## Conclusion

The use of Dixon showed higher sensitivity and AUC for detecting the activity of TAO than T2 mapping. Dixon is expected to further improve the diagnostic accuracy of the activity of TAO.

## Limitation

Firstly, most of the studies included were from Asia, especially China, which may cause research bias due to the largest disease population. Secondly, the EUGOGO CAS score, generated from Europe and America, was based on Caucasian populations. The lower incidence of eyelid redness and swelling in Asians than in Caucasians (5.13%–10.26% vs. 53.5%) may lead to lower sensitivity in the CAS score. Finally, Dixon is an emerging diagnostic method for TAO, and more lines of evidence need to be further collected.

## Data Availability

The original contributions presented in the study are included in the article/**Supplementary Material**. Further inquiries can be directed to the corresponding authors.

## Supplementary material

Supplementary Figure 1 Deeks’ funnel plot.

Supplementary Figure 2 Bivariate boxplot.

Supplementary Figure 3 T2-Multiple univariate meta-regression and subgroup analysis. Prospective design: prodesign; fulverif: partial verification bias; subjdescr: adequate description of study participants

Supplementary Figure 4 Deeks’ funnel plot.

Supplementary Figure 5 Bivariate boxplot.

Supplementary Figure 6 Dixon-Multiple univariate meta-regression and subgroup analysis. Fulverif: partial verification bias; subjdescr.
