# Supplementary figures and images for: The diagnostic performance comparison between T2 mapping and Dixon against the activity of thyroid-associated ophthalmopathy: a systematic review and meta-analysis
Source: Front Endocrinol (Lausanne). 2024 Dec 12;15:1502296. doi: 10.3389/fendo.2024.1502296 (PMC11669502; doi:10.3389/fendo.2024.1502296)

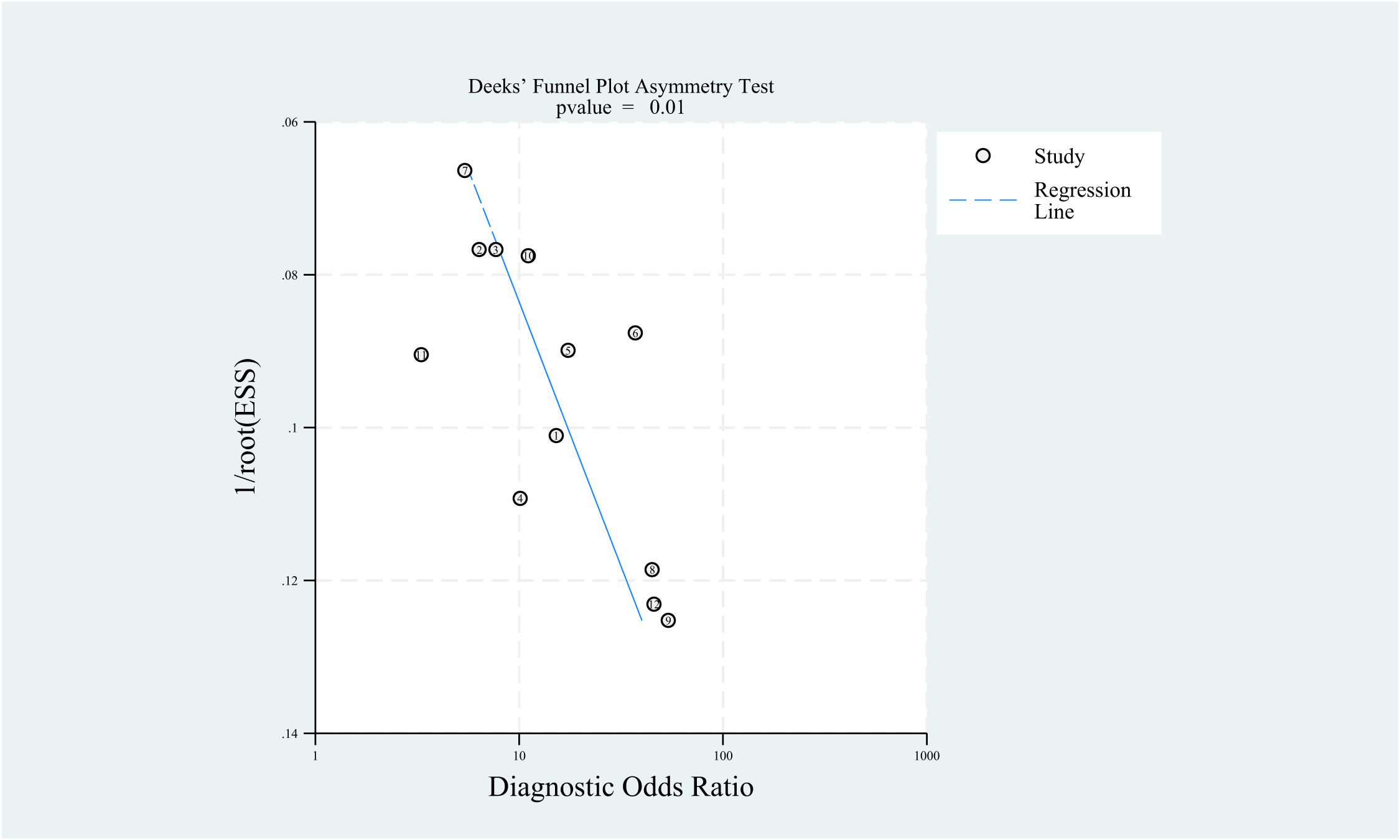

Supplement: Supplementary Figure 1 — Deeks’ funnel plot. [file Image1.tif]

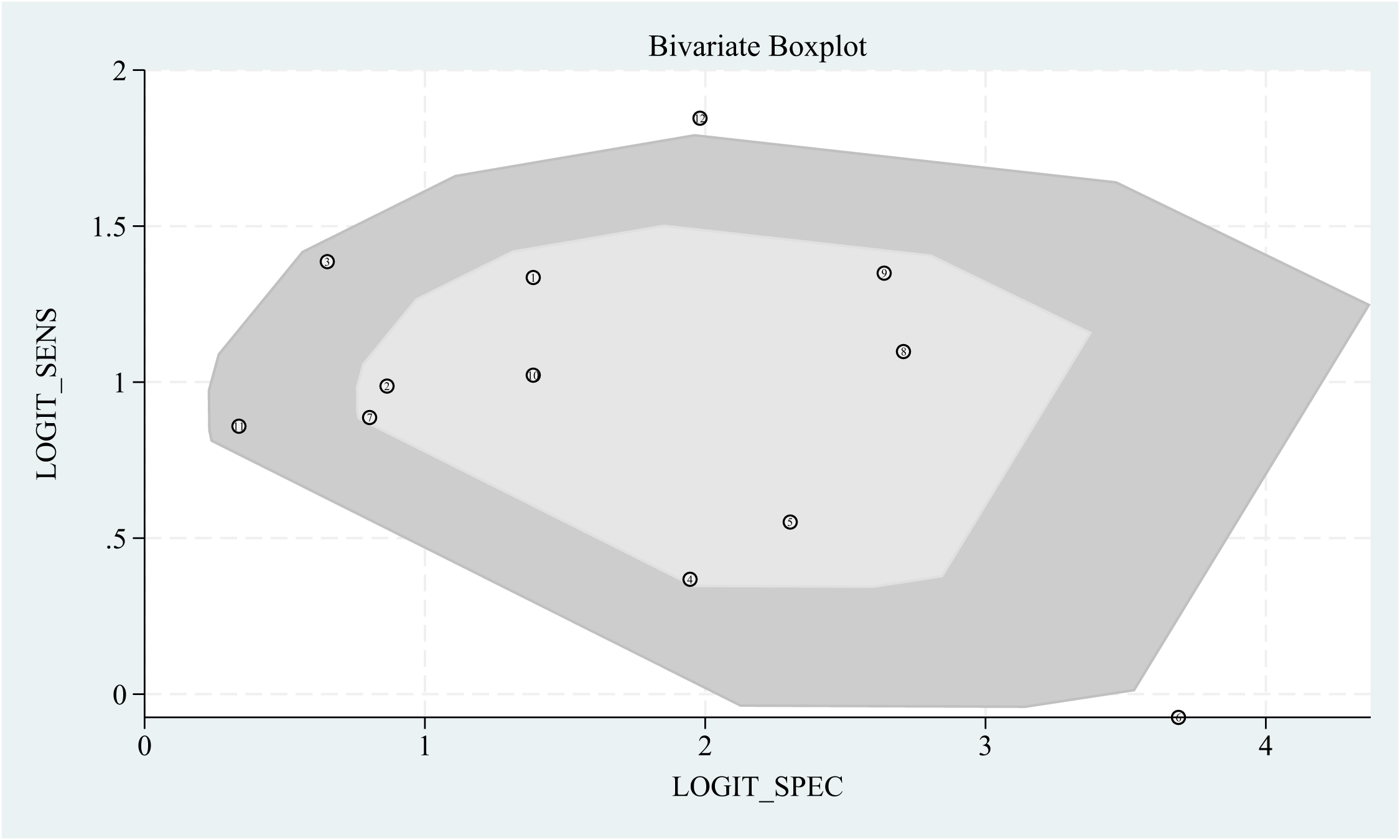

Supplement: Supplementary Figure 2 — Bivariate boxplot. [file Image2.tif]

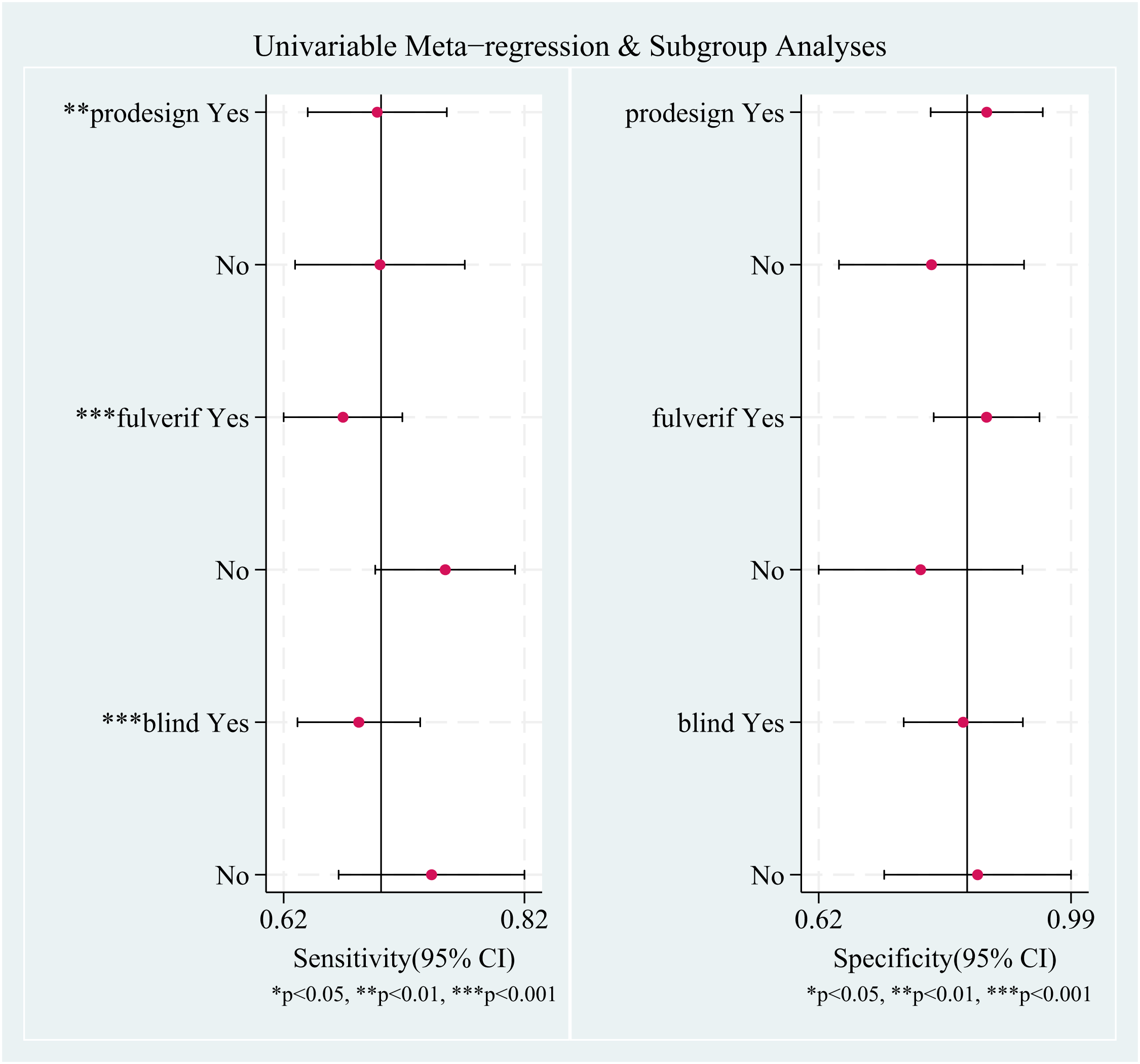

Supplement: Supplementary Figure 3 — T2-Multiple univariate meta-regression and subgroup analysis. Prospective design: prodesign; fulverif: partial verification bias; subjdescr: adequate description of study participants [file Image3.tif]

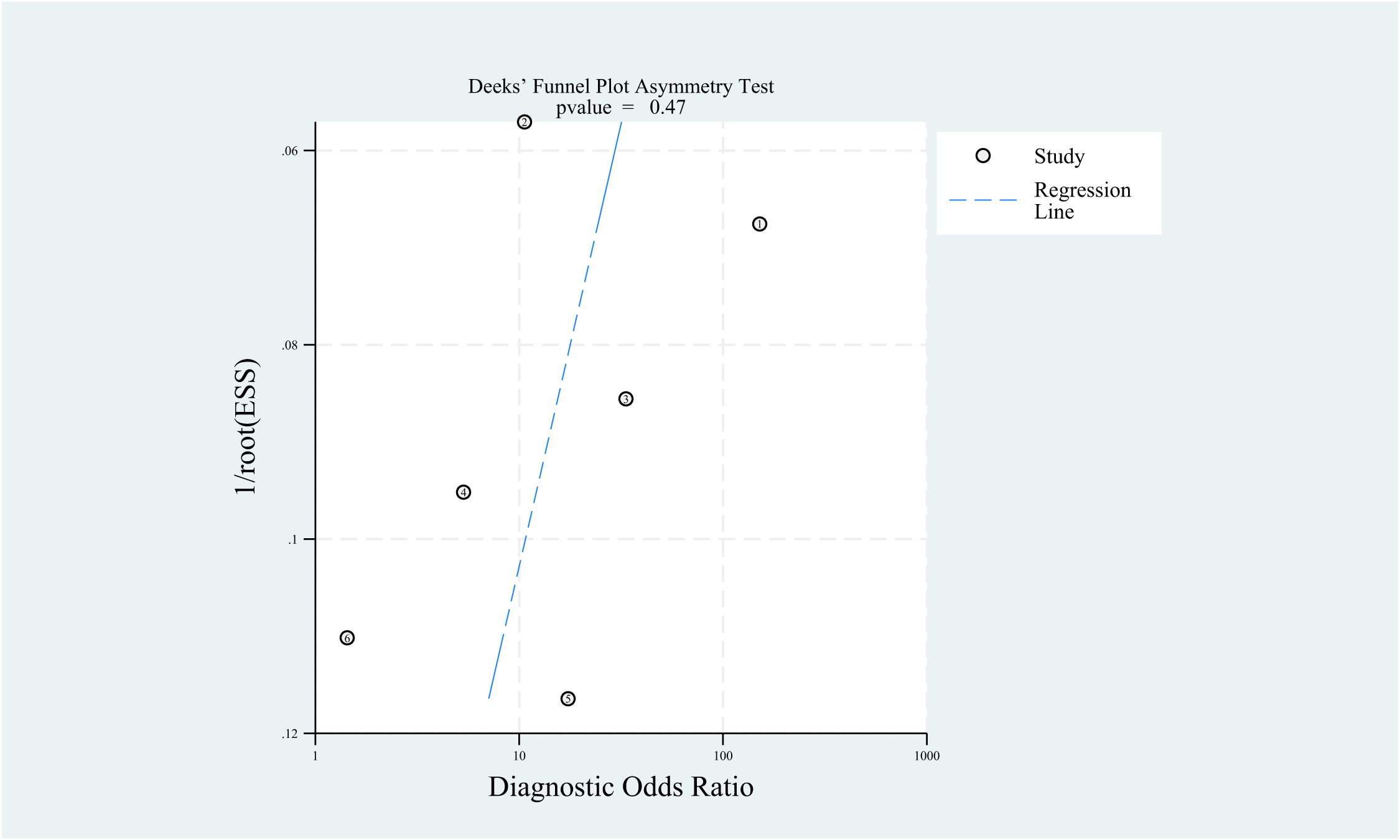

Supplement: Supplementary Figure 4 — Deeks’ funnel plot. [file Image4.tif]

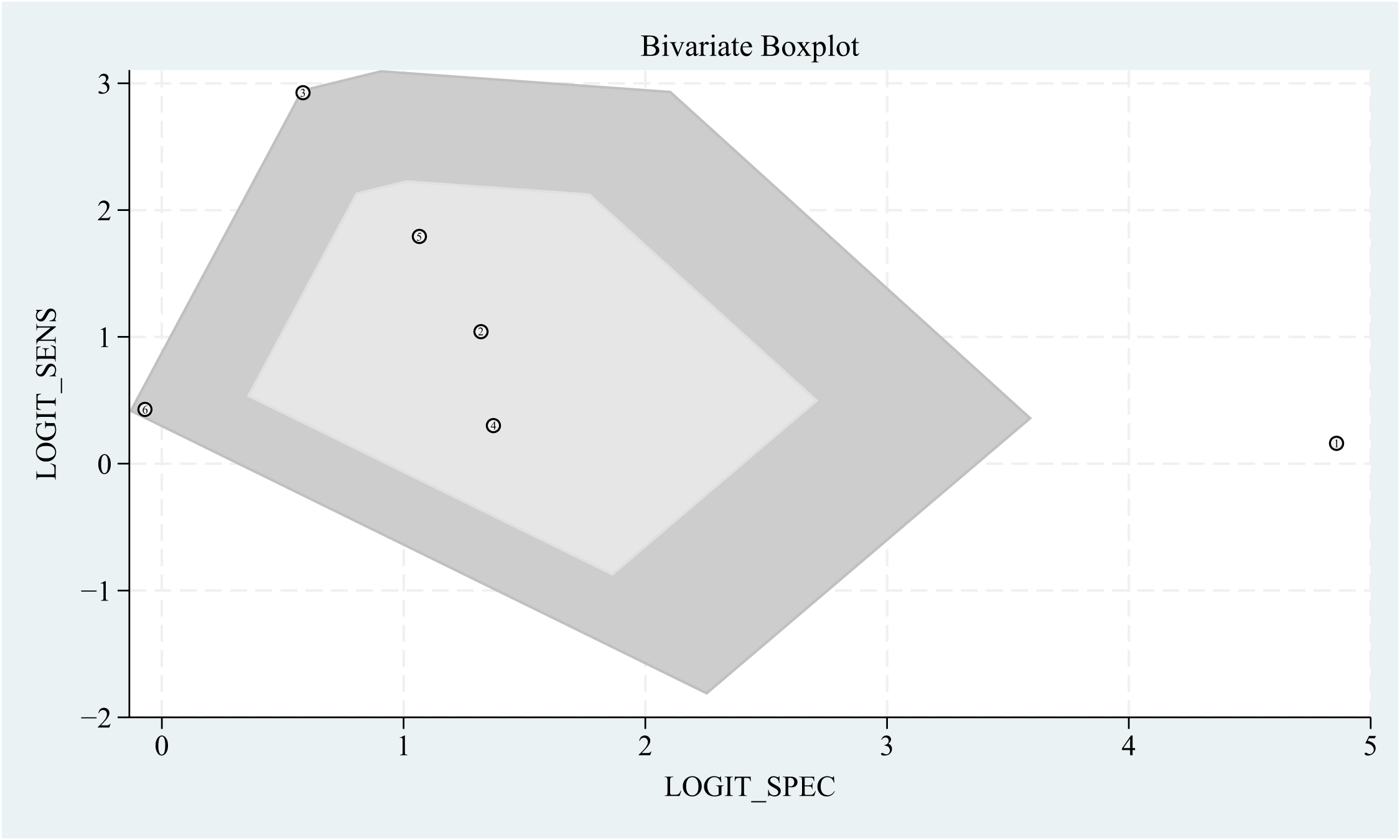

Supplement: Supplementary Figure 5 — Bivariate boxplot. [file Image5.tif]

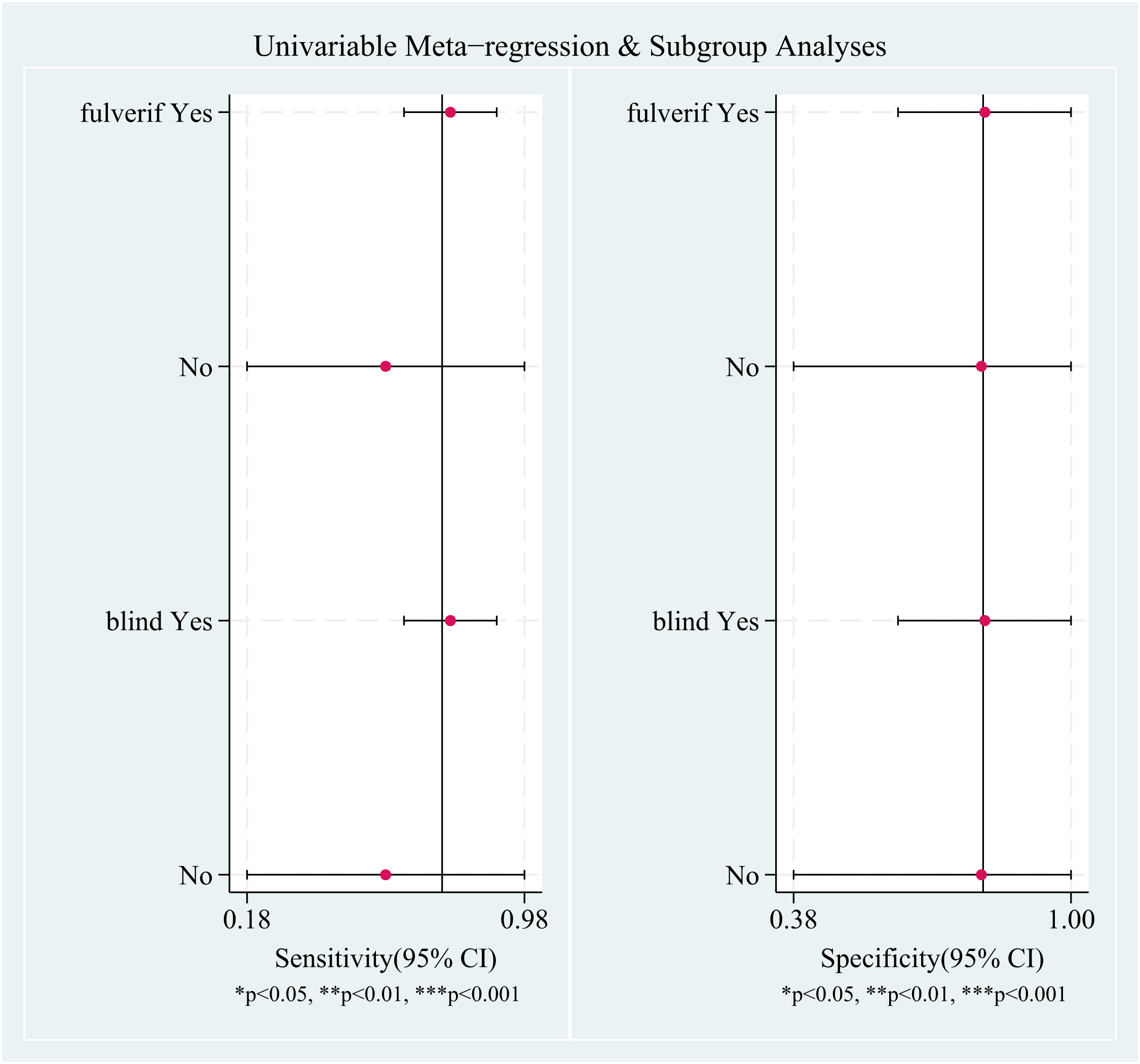

Supplement: Supplementary Figure 6 — Dixon-Multiple univariate meta-regression and subgroup analysis. Fulverif: partial verification bias; subjdescr. [file Image6.tif]
